# The role of ^68^Ga-DOTA derivatives PET-CT in patients with ectopic ACTH syndrome

**DOI:** 10.1530/EC-20-0089

**Published:** 2020-03-26

**Authors:** Filippo Ceccato, Diego Cecchin, Michele Gregianin, Giacomo Ricci, Cristina Campi, Filippo Crimì, Marta Bergamo, Annibale Versari, Carmelo Lacognata, Federico Rea, Mattia Barbot, Carla Scaroni

**Affiliations:** 1 Endocrinology Unit, Department of Medicine DIMED, University-Hospital of Padova, Padova, Italy; 2 Department of Neuroscience DNS, University of Padova, Padova, Italy; 3 Nuclear Medicine Unit, Department of Medicine – DIMED, University-Hospital of Padova, Padova, Italy; 4 Padova Neuroscience Center PNC, University of Padova, Padova, Italy; 5 Nuclear Medicine Unit, Castelfranco Veneto, Italy; 6 Department of Mathematics ‘Tullio Levi-Civita’ DM, University of Padova, Padova, Italy; 7 Radiology Unit, Department of Medicine DIMED, University-Hospital of Padova, Padova, Italy; 8 Nuclear Medicine Unit, Reggio Emilia, Italy; 9 Radiology Department, University-Hospital of Padova, Padova, Italy; 10 Thoracic Surgery Unit, Department of Cardiac, Thoracic and Vascular Sciences, University Hospital of Padova, Padova, Italy

**Keywords:** ectopic Cushing’s syndrome, diagnosis, computed tomography, ^68^Ga-SSTR-PET/CT

## Abstract

**Introduction and aim:**

Ectopic ACTH secretion (EAS) is mostly secondary to thoracic/abdominal neuroendocrine tumours (NETs) or small cell-lung carcinoma (SCLC). We studied the diagnostic accuracy of CT with ^68^Ga-Dota derivatives (^68^Ga-SSTR) PET in localizing ACTH-secreting tumor in patients with EAS.

**Materials and methods:**

^68^Ga-SSTR-PET/CT was performed and compared with the nearest enhanced CT in 18 cases (16 primary and 2 recurrent neoplasms). Unspecific, indeterminate and false-positive uptakes were assessed using conventional imaging, follow-up or histology.

**Results:**

We diagnosed 13 thoracic (9 primary and 2 recurrent bronchial carcinoids, 2 SCLCs) and 1 abdominal (pancreatic NET) tumors. Eight ACTH-secreting tumors were promptly identified at EAS diagnosis (’overt’, four pulmonary carcinoids with two recurrences and two SCLC); six EAS have been discovered during the subsequent follow-up (’covert’, five bronchial carcinoids and one pancreatic NET). At the time of EAS diagnosis, imaging was able to correctly detect the ACTH-secreting tumour in 8/18 cases (6 new diagnosis and 2 recurrences). During the follow-up, six out of initially ten ‘occult’ cases became ‘covert’. At last available follow-up, CT and ^68^Ga-SSTR-PET/CT were able to diagnose 11/18 and 12/18 ACTH-secreting tumours, respectively (11/14 and 12/14 considering only overt and covert cases, respectively). Four cases have never been localized by conventional or nuclear imaging (’occult EAS’), despite an average follow-up of 5 years.

**Conclusion:**

The ^68^Ga-SSTR-PET/CT is useful in localizing EAS, especially to enhance positive prediction of the suggestive CT lesions and to detect occult neoplasms.

## Introduction

Cushing’s syndrome (CS), characterized by excessive endogenous cortisol secretion, is in most cases ACTH-dependent. Corticotropin (ACTH) secretion arises from a pituitary adenoma (Cushing’s disease) or, less frequently, from a non-pituitary neoplasm (ectopic ACTH secretion, EAS) (1, 2, 3, 4). Achieving the goals of CS treatment (to normalize cortisol levels, to reverse the clinical symptoms and to remove the secreting neoplasm) is a challenge, especially in EAS (5, 6).

EAS is defined overt when the ACTH-secreting neoplasm is promptly identified soon after diagnosis of hypercortisolism, covert when the tumour is discovered during a subsequent evaluation or a prolonged follow-up and occult when the ACTH source cannot be detected despite a meticulous and extended follow-up (4, 7). The most common tumours in EAS are thoracic (lung or mediastinal carcinoids, small cell-lung carcinoma SCLC, thymic tumours and medullary thyroid carcinomas) or abdominal neoplasms (islet cell tumours of the pancreas, pheochromocytoma, gastrointestinal carcinoids) (8, 9, 10). In the larger series reported, patients with occult EAS represent 8–32% of described cases (7, 9, 11, 12).

Once EAS is suspected, conventional imaging is usually the first approach to localize the tumor: the overall reported sensitivity is 66% for CT and 51% for magnetic resonance (MR) in overt EAS (10). However, 30% of EAS could be detected only during follow-up: CT is able to detect ACTH-secreting neoplasm in 44% of covert EAS (10). In patients with negative CT and/or MR, nuclear medicine improves the sensitivity of conventional radiology: a positive finding is described in 67% of ^111^In-Octreoscan and in 60% of ^18^F-fluorodeoxyglucose (^18^F-FDG) PET/CT (10). Furthermore, almost 75% of patients with initial occult EAS at conventional imaging became covert with nuclear imaging, achieving a high sensitivity with PET/CT using ^68^Ga-conjugated somatostatin receptor targeting peptide (^68^Ga-SSTR-PET/CT). In 2016 Goroshi *et al*. compared, in a consecutive series of 12 patients, the diagnostic accuracy of conventional (contrast-enhanced CT) and nuclear (^68^Ga-SSTR-PET/CT) imaging. CT detected 90% of NETs in overt EAS, whereas ^68^Ga-SSTR-PET/CT identified 70% of cases, without reporting false-positive imaging, resulting useful to increase the specificity of the suggestive CT-positive lesions. In this series, the only EAS not detected with conventional imaging remained occult also after the ^68^Ga-SSTR-PET/CT (13). In a recent multicenter study, Wannachalee *et al*. reported in 28 patients that ^68^Ga-SSTR-PET/CT is sensitive to detect primary and metastatic neoplasms in EAS and to identify occult tumours after other type of imaging (in 65% of cases), achieving a significant clinical impact in the diagnostic-therapeutic management in the majority of patients (14).

ACTH-secreting neoplasms present several receptors, especially SSTRs, the target of theranostic somatostatin-based diagnosis (with octreoscan ^68^Ga-SSTR-PET/CT (10, 13, 14)) or treatment (with somatostatin analogs (15, 16)). In EAS, excessive glucocorticoid levels can directly down-regulate SSTR expression, especially type 2, thus resulting in a possible false-negative ^68^Ga-SSTR-PET/CT in EAS patients with active hypercortisolism that could revert after normalization of cortisol levels achieved with steroidogenesis inhibitors (17).

The aims of our study were to study the diagnostic accuracy of ^68^Ga-SSTR-PET/CT in a monocentric series of consecutive patients with EAS and to consider cortisol levels according to imaging.

## Materials and methods

### Patients

Patients with EAS were enrolled at the Endocrinology Unit of Padova. The diagnosis of CS was confirmed by at least two impaired results using first-line screening tests: elevation of 24-h urinary free cortisol (UFC), absent serum cortisol suppression (<50 nmol/L) after overnight 1 mg dexamethasone suppression test (DST) and loss of circadian salivary cortisol rhythm.

The diagnosis of ACTH-dependent CS derived from the finding of normal or elevated morning ACTH levels (>10 ng/L). EAS was suspected on the basis of second-line tests (absence of ACTH increase after CRH stimulation test, unsuppressed serum cortisol after 8-mg DST, increased urinary cortisol/cortisone ratio) previously described (18, 19). Bilateral inferior petrosal sinus sampling (BIPSS) was performed in patients with at least one discordant test; a central/peripheral ACTH ratio <2 in basal conditions and <3 at after CRH stimulus allowed us to rule out Cushing’s disease.

We considered overt EAS in patients with an ACTH-secreting tumour discovered early after CS diagnosis, covert cases those when the discovery of ACTH-secreting tumour was performed at least 6 months after CS diagnosis and occult those patients without the identification of the ACTH source. EAS was confirmed by histological finding of positive ACTH immunostaining in all overt and covert cases.

Serum cortisol and ACTH levels were measured by chemiluminescence immunoassay (Immulite 2000, Siemens Healthcare). Urinary cortisol and cortisone were assessed by liquid chromatography with tandem mass spectrometry (LC-MS/MS) with an Agilent HPLC series 1200 coupled with an Agilent 6430 triple quadrupole mass spectrometer equipped with an Electrospray Ionization source, operating in positive ion mode (Agilent Technologies) (20). Loss of circadian rhythm was measured with late night salivary cortisol (LNSC). Salivary cortisol, collected with Salivette device (Sarted, Numbrecht, Germany), was measured with radio-immunometric assay until 2014 (Radim, Rome, Italy, previously reported (21)), then with LC-MS/MS, as previously described (22).

Clinical data were collected in the web-based database of the University-Hospital of Padova, used as an electronic Case Report/Record Form (eCRF). Ethics Committee of Padova University-Hospital approved the study protocol, and all patients gave written informed consent.

### Conventional and nuclear imaging

From the cohort of EAS cases (*n* = 30), we selected only those patients who performed a ^68^Ga-SSTR-PET/CT at baseline (considered as the initial CS diagnosis) in two Italian referral centres. In occult cases, a whole-body CT was performed 6 months after diagnosis and then yearly, and a ^68^Ga-SSTR-PET/CT was repeated every 18–24 months or in case of positive CT findings.

We considered 16 patients, 9 females and 7 males, with mean follow-up upto March 2019 of 5 ± 2.6 years. We collected 30 acquisitions (at baseline, and during follow-up in occult cases), 25 using somatostatin analog DOTATOC and 5 with DOTANOC. The standard uptake value (SUV) of the reported lesions was extracted and the number of true-positive, false-negative and false-positive images were calculated considering conventional imaging, patient’s history or final histology as confirmation. We compared each ^68^Ga-SSTR-PET/CT with the temporal nearest conventional imaging (CT or MR) in order to confirm the number of true-positive, false-negative and false-positive lesions. We also collected morning serum cortisol and ACTH, UFC and LNSC the week before ^68^Ga-SSTR-PET/CT imaging.

In Castelfranco Veneto, a GE Discovery 710 tomograph was used with 120 kEv, 80–90 mA (modulable), 3.75 mm slices thickness and 4 min/bed acquisition time CT parameters. The PET reconstruction matrix was 256 × 256, the injected activity was 2 MBq/kg (in any case not less than 100–110 MBq) and the waiting time between injection and acquisition was 60 ± 10 min. The ^68^Ga was obtained through a ^68^Ge/^68^Ga ITG generator (ITM Group, Schwalmtal, Germany) with 1.85 GBq activity, then labeled to DOTATOC through a synthesis module. In Reggio Emilia PET/CT was performed with a hybrid scanner (Discovery STE; GE Healthcare) with a sensitivity equal to 9.365 cps/kBq, according to National Electrical Manufacturers Association 2001. The CT attenuation correction acquisition parameters were 120-kV voltage, 80-mA tube current and 3.75-mm slice thickness. Images were reconstructed using the 3D ordered-subsets expectation maximization, with a 256 × 256 matrix and a voxel size of 2.73 × 2.73 × 3.27 mm^3^. ^68^Ga was obtained from a commercially available ^68^Ge/^68^Ga generator (Ciclotron, Napa, CA, USA) with a nominal activity of 1.85 GBq. The administered dose of ^68^Ga DOTATOC was 2 MBq/kg and the uptake time was 60 ± 10 min after tracer injection; PET images lasted for 5 min/bed position.

### Statistical analyses

Proportions and rates were calculated for categorical data; continuous data were reported as means and s.e. or median and interquartile range (IQR). We correlated the SUV^Max^ of each lesion with the corresponding hormonal levels (morning serum cortisol, ACTH, UFC and LNSC). The database was managed and statistical analysis performed by SPSS 17 software package for Windows (SPSS, Inc.). Significance level was set as a *P* < 0.05 for all tests.

## Results

### Patients

All patients presented increased UFC levels, impaired cortisol rhythm, unsuppressed serum cortisol after 1-mg DST and normal-increased ACTH levels. Considering second-line tests for the diagnosis of ACTH-dependent CS, in 13 out of 14 cases the response of ACTH or cortisol to CRH test was absent, 11 out of 13 patients did not achieve sufficient cortisol suppression after 8-mg DST and their urinary cortisol to cortisone ratio was increased in 8 out of 11 patients. BIPSS (performed in ten patients) excluded a pituitary gradient in all cases. Eleven out of fourteen patients had an elevation of chromogranin A, 5/15 of NSE and 5/13 of CYFRA.

Overweight or obesity was found in 9 patients, weight loss in 3, hypertension in 15, diabetes mellitus or impaired fasting glucose in 14, hypokalaemia in 15, osteoporosis or fracture in 13, dyslipidaemia in 9, psychological disorders in 11, proximal muscular atrophy in 10, skin thinning and bruise in 5 cases and skin pigmentation in 1 patient; hirsutism was observed in 2 female patients.

### Diagnostic accuracy of conventional imaging and 68Ga-SSTR-PET/CT

**Figure 1:**
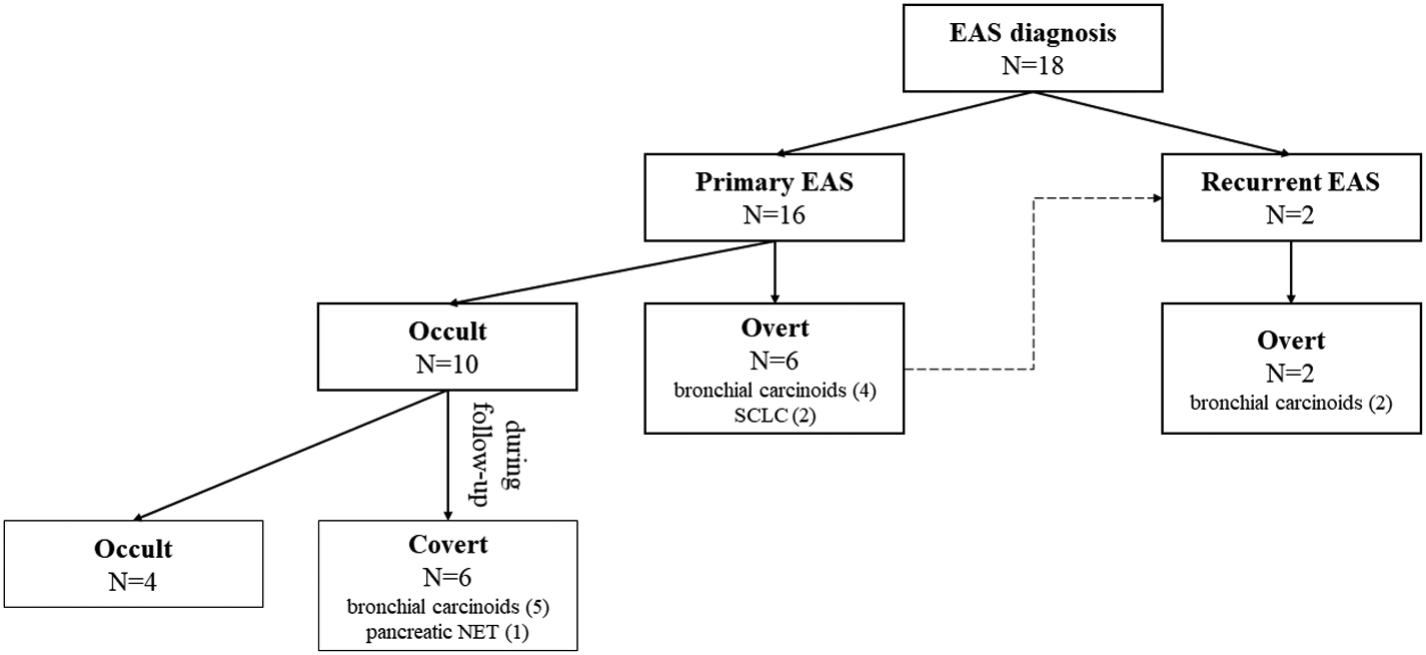
Timeline of EAS diagnosis: eight overt (two recurrences), six covert and four occult cases.

Considering all patients, at baseline eight EAS were overt (six new diagnosis and two recurrences of previous overt cases in patients with lung carcinoids) and ten were occult, as summarized in Fig. 1.

Regarding overt EAS, all cases were localized by imaging methods within the first 6 months from the diagnosis of CS. All overt EAS were thoracic tumors: six patients with bronchial carcinoids (four new diagnosis and two recurrences) and two with SCLC. Excluding the two recurrences, in the six patients with overt EAS, at CS diagnosis, CT identified 4/6 and ^68^Ga-SSTR-PET/CT 6/6 ACTH-secreting tumours: in two cases ^68^Ga-SSTR-PET/CT was the first technique able to identify the primary tumour (CT was not conclusive before the nuclear medicine imaging, and confirmed the suspicion only after PET/CT). In the other four patients, ^68^Ga-SSTR-PET/CT was performed after the positive CT and confirmed the neuroendocrine origin of the tumors. Both recurrent cases were correctly discovered with CT, one relapse was not detected with ^68^Ga-SSTR-PET/CT.

Considering the initial ten occult EAS, six out of ten were localized only after a careful follow-up (median 2 years), therefore were considered covert EAS: five were bronchial carcinoids and one pancreatic NET. One of them was found during CT and it never showed a pathological uptake of ^68^Ga-SSTR; another tumour was not initially seen by conventional radiology techniques, but the lesion was identified after positive ^68^Ga-SSTR-PET/CT. In those patients with overt and covert EAS (eight overt and six covert ACTH-secreting tumours), CT and ^68^Ga-SSTR-PET/CT identified neoplasms in 11/14 and 12/14, respectively (as summarized in Table 1).

**Table 1:** Description of cases, EAS diagnosis and type, conventional imaging and ^68^Gallium-SSTR-PET/CT true-positives uptakes (SUV^max^ in brackets).

Case, age, gender	Age of EAS diagnosis	Timing and type of EAS	Conventional imaging	68Ga-DOTA PET/CT (SUV^Max^)	Conventional imaging	68Ga-DOTA PET/CT (SUV^Max^)
			Baseline		Follow-up	
1a, 57, F	50	Overt: lung carcinoid	CT: ✗	✓ (2.57)	n.a.	n.a.
1b, 57, F	51	Overt: nodal recurrence	n.a.	n.a.	CT: ✓	✓ (4.65)
2, 76, F	73	Occult (died 6 years after EAS diagnosis)	CT: ✗	✗	n.a.	n.a.
			MR: ✗			
3, 77, M	72	Overt: SCLC	CT: ✓	✓ (3.1)	CT: ✗	n.a.
4, 68, M	67	Overt: lung carcinoid	CT: ✓	✓ (4.7)	CT: ✗	n.a.
5, 82, M	68	Occult	CT: ✗	✗	CT: ✗	✗
6, 62, F	53	Occult (died 9 years after EAS diagnosis)	CT: ✗	✗	CT: ✗	✗
7, 78, F	72	Occult	CT: ✗	✗	CT: ✗	✗
			MR: ✗	✗	CT: ✗	
8, 62, F	53	Covert: lung carcinoid (2 years)	CT: ✗/✓	✗	CT: ✓	✗
			MR: ✗		MR: ✓	
9, 42, M	32	Covert: lung carcinoid (3 years)	CT: ✗	✗	CT: ✓	✓ (3.4)
10, 73, F	66	Overt: SCLC	CT: ✓	✓ (10)	CT: ✗	n.a.
11, 66, F	57	Covert: lung carcinoid (2 years)	CT: ✗	✗	CT: ✓	✓ (3.8)
			MR: ✗		MR: ✗	
12, 33, M	23	Covert: lung carcinoid (2 years)	CT: ✗	✗	CT: ✓	✓ (5)
			MR: ✗		MR: ✓	
13, 85, F	84	Overt: lung carcinoid (died 2 years after diagnosis)	CT: ✓	✓ (16.7)	CT: ✗	✗
14, 71, M	58	Covert: pancreatic NET (3 years)	CT: ✗	✗	CT: ✓	✓ (50)
15a, 67, F	65	Overt: lung carcinoid	CT: ✗	✓ (4.3)	n.a.	n.a.
15b, 67, F	67	Overt: nodal relapse	n.a.	n.a.	CT: ✓	✗
16, 46, M	42	Covert: lung carcinoid (6 months)	CT: ✗	✗	CT: ✗	✓ (7.9)
			MR: ✗			

We reported the first positive conventional imaging or ^68^Ga-DOTA PET/CT in case of overt and covert EAS (the time span from occult to covert is described in brackets) at EAS diagnosis (baseline) or during follow-up (indicating which imaging technique was positive to localize the tumour).

EAS, ectopic ACTH secretion; NET, neuroendocrine tumour; SCLC, small cell lung cancer; ✓, positive; ✗, negative; ✗/✓, indeterminate lesion, confirmed as the source of ACTH secretion in the follow-up.

At last follow-up visit available, in four cases neither conventional imaging nor ^68^Ga-SSTR-PET/CT were able to find the ACTH-secreting tumors, thus remaining an occult EAS (median follow-up of 5 years). Therefore, the final diagnostic accuracy was 11/18 and 12/18 for CT and ^68^Ga-SSTR-PET/CT, respectively. In these four occult cases, the alternative diagnosis of a Cushing’s disease was ruled out because BIPSS excluded a pituitary ACTH secretion.

We observed a weak inverse relationship between SUV^Max^ and cortisol secretion: increase of SUV^Max^ was poorly correlated with a decrease of serum cortisol (y = -0.0108x + 19.886, correlation coefficient *r* = 0.24) and UFC (y = -0.0022x + 14.995, *r* = 0.28; both *P* > 0.05), probably secondary to the low number of subjects considered.

### False-positive and false-negative uptakes of nuclear imaging

All images were reviewed by experienced radiologists and nuclear medicine physicians.

**Figure 2:**
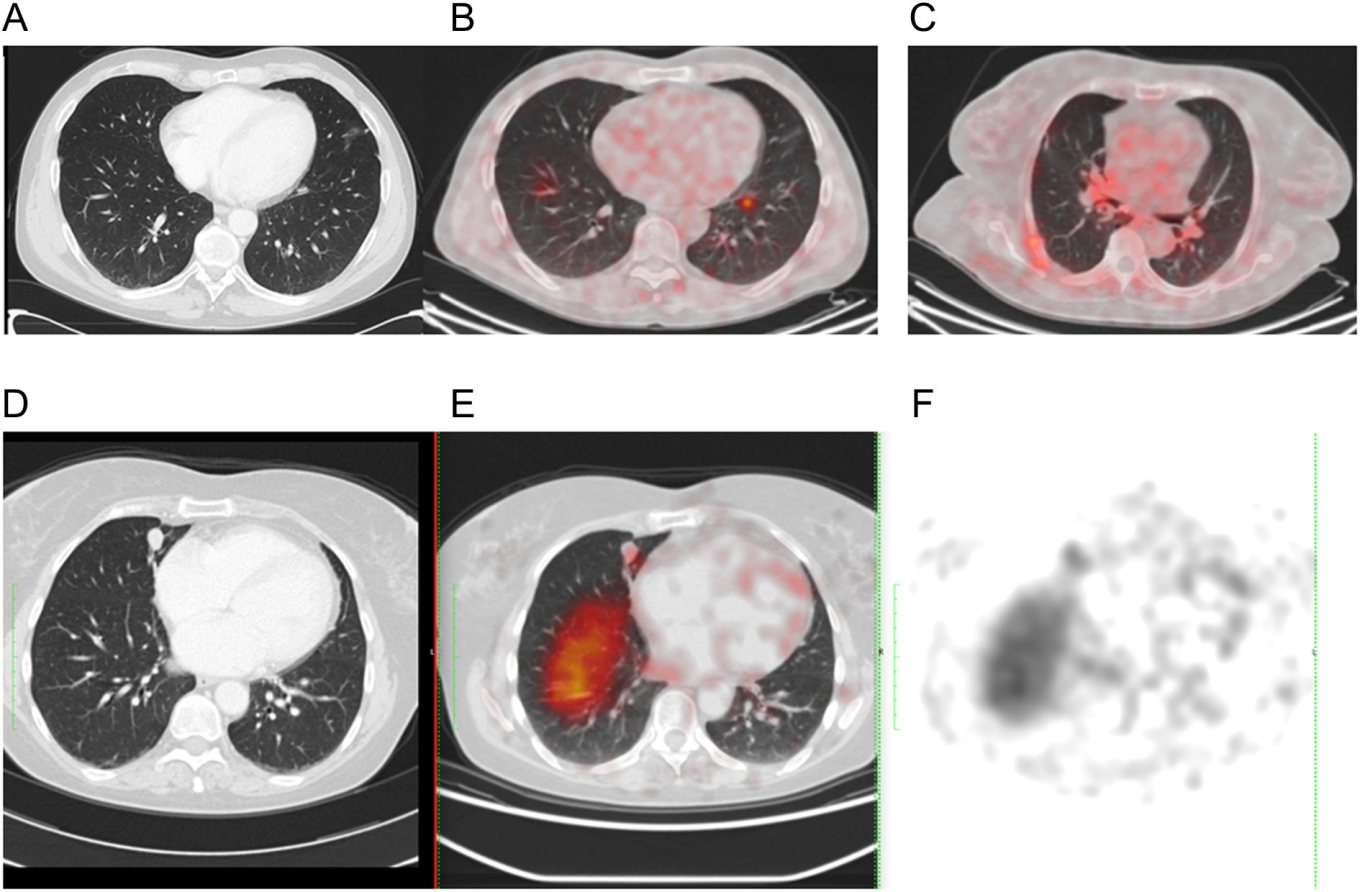
CT and ^68^Ga-SSTR-PET/CT images. Images of patient 9: (A) CT and (B) ^68^Ga-SSTR-PET/CT. The bronchial carcinoid has been individuated in the CT after the discovery of pulmonary uptake in the ^68^Ga-SSTR-PET/CT. Images of patient 15: (C) false-positive slight uptake (chest wall inflammation) after the first surgery for EAS (more than 12 months before). Images of patient 8: (E) the pathological ^68^Ga-SSTR-PET/CT uptake was not localized correctly initially ((D) CT image) because of its right inferior pulmonary localization and the overlap with liver uptake, due respiratory motion during acquisition of PET and CT. (F) emission tomography image.

A case of bronchial carcinoid with bone secondary lesions was only seen at ^68^Ga-SSTR-PET/CT. A ‘covert’ patient with a positive uptake of ^68^Ga-SSTR-PET/CT at the primary lesions had a cervical nodal relapse negative to ^68^Ga-SSTR-PET/CT but positive to ^18^F-FDG PET, probably because of dedifferentiation of tumour cells and reduced expression of somatostatin receptors. In another case, hepatic metastasis at CT was not identified at ^68^Ga-SSTR-PET/CT because of physiological inhomogeneous uptake of the pharmaceutical in the liver. A pathological ^68^Ga-SSTR-PET/CT uptake was overseen at PET/CT because of its right inferior pulmonary localization and the accidental overlap with liver uptake (see Fig. 2, the first PET/CT was reported as negative).

As described in Table 2, the number of indeterminate or false-positive results of ^68^Ga-SSTR-PET/CT was not a minor concern. Incidental uptakes, caused by pharmacokinetic of ^68^Ga-SSTR in liver, spleen, kidney, ureter and bladder or by SSTR expression in pancreas, bowel, thyroid and pituitary gland were recognized as unspecific. Adrenal gland was the most frequent site of inappropriate uptake, with greater SUV^Max^. ^68^Ga-SSTR uptakes caused by inflammatory diseases (gastritis, previous surgical approach, reactive lymph nodes, arthrosis) were also correctly interpreted as non-neoplastic. False-positive cases required further investigations, such as CT for a vertebral haemangioma or MR for a pancreatic lesion (an intraductal papillary mucinous neoplasm).

**Table 2:** Non-specific (NS) and/or false positive (FP) uptakes of ^68^Gallium-SSTR-PET/CT (SUV^max^ is reported in brackets).

Case	Head, neck, thorax	Abdomen	Lymph nodes	Skeleton	Explanation
1, a	Right lung (3, NS)	Adrenal (4.8, NS), stomach (NS)	Axilla (1.2, NS), groin (1.6, NS)	Left hip, spine L5–S1 (4, FP)	Gastric inflammation (confirmed with gastric endoscopy and biopsy), reactive nodes (disappeared in the follow-up), arthrosis, hip uptake disappeared during follow-up. No adrenal node.
2	Thyroid (5.5, NS)	Pancreas (9.3, FP), adrenal (16.7, NS)		Skull (31.7, FP)	Not suggestive of NET (no adrenal or pancreatic nodes in conventional imaging, no thyroid node during neck ultrasound, ACTH-secreting tumour excluded with autopsy), bone inflammation.
3		Adrenal (13, NS)			Not suggestive (no adrenal lesions at CT and MR).
7	Thyroid (6.8, NS)	Pancreas (8.5, FP), adrenal (26.2, FP)			Not suggestive (no adrenal or pancreatic lesions at CT).
9	Mediastinum (1.5, NS)		Groin (2)	Spine D3 (3.8, NS)	Spine haemangioma (pathognomonic polka-dot sign), reactive nodes.
13		Adrenals (17.2, NS)			Not suggestive (no adrenal lesions at CT and MR).
14	Thyroid (10, FP)				Not suggestive (no thyroid node at neck ultrasound).
15, b		Duodenum (8.4, FP)		Ribs (4, NS)	Physiologic pancreatic-duodenal uptake and absence of neoplasms with MR and endoscopic ultrasonography, chest wall inflammation after surgery.
16		Pancreas (15.46, FP)		Spine D5–D6 (5.6, NS), right femur (3.38, NS)	Focal areas of uptake disappeared at follow-up, no pancreatic lesion at CT and MR (false-positive pancreatic uptake: it was a lung carcinoid).

## Discussion

The proper localization of the ACTH-secreting tumor in patients with EAS is crucial not only to indicate the surgical treatment but also to reduce cortisol-related comorbidities and to minimize the risk of disease progression (1, 5, 7, 8). In this work we described the systematic use of ^68^Ga-SSTR-PET/CT in consecutive patients with EAS, considering also the role of hypercortisolism.

We reported 14 patients with overt and covert EAS: 11 patients with bronchial carcinoids (9 new cases and 2 recurrences), 2 SCLCs and 1 case of pancreatic NET. A systematic review of EAS in 2015 found a lower sensitivity of CT than ^68^Ga-SSTR-PET/CT (10), but other studies presented discordant results (13). The more common use of CT in EAS may be explained by the prevalent distribution of neoplasms in the thorax, where MR is problematic because of its less resolution in the lung parenchyma, due to respiratory and cardiac artefacts (7). In our series, in some of the cases presented, tumours could be recognized at CT only after the discovery of a pathological uptake of ^68^Ga-SSTR-PET/CT. The number of tumours that are not localized even after a long follow-up, ‘occult’ cases, can range from 9 to 50% depending of the series (4, 7, 13, 14, 23). In our study, a quarter of patients with EAS remained occult at the last available follow-up, as recently reported in a series with similar diagnostic approach (14). Nevertheless, in this series they collected patients in three referral centers, thus a bias of different management cannot be excluded *a priori*.

^68^Ga-SSTR-PET/CT was previously suggested to confirm the discovery of EAS because of its lower false-positive rate (13, 14), while other authors found a greater false-positive rate and suggested the nuclear imaging when CT and MR are both negative (10). In our series ^68^Ga-SSTR-PET/CT revealed a not-negligible number of unspecific uptakes, whose interpretation required a careful revision. Even reported physiologic uptakes may be confusing in certain cases and prevent the proper localization of EAS, as it happened in patient number 3 in our series, with hepatic metastasis emerged at CT. Particular attention should be paid to the adrenal uptake that is usually physiologic, but could contemporarily hide an adrenal ACTH secretion (EAS has been reported in patients with pheochromocytoma (24)), leading to a therapeutic delay (14). Moreover, adrenals are often hyperplastic because of chronic ACTH stimulus and a great incidence of adrenal nodes has been correlated to ACTH-dependent CS (25). To discriminate these possibilities, the combination of different imaging may be useful, such as CT/MR and ^18^F-DOPA-PET (10, 24). In our series, unspecific adrenal uptakes of ^68^Ga-DOTA showed often a greater SUV^Max^ than the primary ACTH-secreting tumor itself, because 1–5 SSTRs are widely expressed in adrenals (26, 27). A PET/MR approach using MR sequences and ^68^Ga-SSTR-PET could probably be a reasonable choice in this scenario.

The challenging interpretation of ^68^Ga-SSTR-PET/CT uptakes points to the importance of a careful investigation of medical history, especially aimed at discovering possible inflammatory states, such as chronic gastritis, thyroiditis, articular inflammation and previous surgical access (28). Moreover, a close collaboration between different specialists, in particular between radiologists, nuclear medicine physicians and endocrinologists, is warranted. The combination with conventional imaging (CT/MR/ultrasound) could clarify the nature of unspecific uptakes, as a vertebral hemangioma that cause osteoblast activity: in such case, the ^68^Ga-SSTR-PET/CT positive imaging has been reconsidered as false positive due to the pathognomonic aspect at conventional imaging (29).

Nuclear and conventional imaging should be repeated during the follow-up, not only in occult cases. In our series, the sensitivity increased during the follow-up and six out of ten EAS were localized after the diagnosis of occult EAS (changing the state from occult to covert cases). In occult EAS, ^68^Ga-SSTR-PET/CT is a sensitive choice (10). In the meantime, the medical control of the cortisol excess, or adrenalectomy in extreme cases, can influence the diagnostic accuracy of ^68^Ga-SSTR-PET/CT because of downregulation of SSTR by high cortisol level (17, 30, 31). We observed that the reduction of cortisol levels was weakly related to increased SUV^Max^. A relationship could exist; however, our results did not reach statistical significance (considering the small sample size, a limit in every monocentric study about EAS, caused by the rarity of the disease).

Beside strengths, our work presents some limitations. First, the number of subjects enrolled. Second, the design of the study (observational, open and not randomized). Moreover, a prospective study reporting the results of ^68^Ga-SSTR-PET/CT in the same patient under hypercortisolism and after CS control is warranted.

To conclude, ^68^Ga-SSTR-PET/CT is useful in the clinical management of patients with EAS, especially combined with CT. However, it presents a considerable number of indeterminate/false-positive images that need a careful interpretation.

## Declaration of interest

The authors declare that there is no conflict of interest that could be perceived as prejudicing the impartiality of the research reported.

## Funding

This study did not receive any specific grant from any funding agency in the public, commercial or not-for-profit sector.

## Research involving human participants and patient consent

Informed consent has been obtained.

## Data availability statement

Data are available on request due to local (academic) restrictions.

## References

[1] Arnaldi G Angeli A Atkinson AB Bertagna X Cavagnini F Chrousos GP Fava GA Findling JW Gaillard RC Grossman AB, Diagnosis and complications of Cushing’s syndrome: a consensus statement. Journal of Clinical Endocrinology and Metabolism 2003 88 5593–5602. (10.1210/jc.2003-030871) 14671138

[2] Nieman LK Biller BM Findling JW Newell-Price J Savage MO Stewart PM Montori VM The diagnosis of Cushing’s syndrome: an Endocrine Society Clinical Practice Guideline. Journal of Clinical Endocrinology and Metabolism 2008 93 1526–1540. (10.1210/jc.2008-0125) 18334580 PMC2386281

[3] Boscaro M Arnaldi G Approach to the patient with possible Cushing’s syndrome. Journal of Clinical Endocrinology and Metabolism 2009 94 3121–3131. (10.1210/jc.2009-0612) 19734443

[4] Alexandraki KI Grossman AB The ectopic ACTH syndrome. Reviews in Endocrine and Metabolic Disorders 2010 11 117–126. (10.1007/s11154-010-9139-z) 20544290

[5] Nieman LK Biller BM Findling JW Murad MH Newell-Price J Savage MO Tabarin A & Endocrine Society. Treatment of Cushing’s syndrome: an Endocrine Society clinical practice guideline. Journal of Clinical Endocrinology and Metabolism 2015 100 2807–2831. (10.1210/jc.2015-1818) 26222757 PMC4525003

[6] Ceccato F Barbot M Zilio M Albiger N Mantero F Scaroni C Therapeutic strategies for Cushing’s syndrome: an update. Expert Opinion on Orphan Drugs 2015 3 45–56. (10.1517/21678707.2015.991714)

[7] Isidori AM Kaltsas GA Pozza C Frajese V Newell-Price J Reznek RH Jenkins PJ Monson JP Grossman AB Besser GM The ectopic adrenocorticotropin syndrome: clinical features, diagnosis, management, and long-term follow-up. Journal of Clinical Endocrinology and Metabolism 2006 91 371–377. (10.1210/jc.2005-1542) 16303835

[8] Isidori AM Lenzi A Ectopic ACTH syndrome. Arquivos Brasileiros de Endocrinologia e Metabologia 2007 51 1217–1225. (10.1590/s0004-27302007000800007) 18209859

[9] Ilias I Torpy DJ Pacak K Mullen N Wesley RA Nieman LK Cushing’s syndrome due to ectopic corticotropin secretion: twenty years’ experience at the National Institutes of Health. Journal of Clinical Endocrinology and Metabolism 2005 90 4955–4962. (10.1210/jc.2004-2527) 15914534

[10] Isidori AM Sbardella E Zatelli MC Boschetti M Vitale G Colao A Pivonello R & ABC Study Group. Conventional and nuclear medicine imaging in ectopic Cushing’s syndrome: a systematic review. Journal of Clinical Endocrinology and Metabolism 2015 100 3231–3244. (10.1210/JC.2015-1589) 26158607 PMC4570166

[11] Salgado LR Fragoso MC Knoepfelmacher M Machado MC Domenice S Pereira MA de Mendonça BB Ectopic ACTH syndrome: our experience with 25 cases. European Journal of Endocrinology 2006 155 725–733. (10.1530/eje.1.02278) 17062889

[12] Hernández I Espinosa-de-los-Monteros AL Mendoza V Cheng S Molina M Sosa E Mercado M Ectopic ACTH-secreting syndrome: a single center experience report with a high prevalence of occult tumor. Archives of Medical Research 2006 37 976–980. (10.1016/j.arcmed.2006.05.015) 17045113

[13] Goroshi MR Jadhav SS Lila AR Kasaliwal R Khare S Yerawar CG Hira P Phadke U Shah H Lele VR, Comparison of 68Ga-DOTANOC PET/CT and contrast-enhanced CT in localisation of tumours in ectopic ACTH syndrome. Endocrine Connections 2016 5 83–91. (10.1530/EC-16-0010) 27006371 PMC5002954

[14] Wannachalee T Turcu AF Bancos I Habra MA Avram AM Chuang HH Waguespack SG Auchus RJ The clinical impact of [(68) Ga]-DOTATATE PET/CT for the diagnosis and management of ectopic adrenocorticotropic hormone – secreting tumours. Clinical Endocrinology 2019 91 288–294. (10.1111/cen.14008) 31066920 PMC6689243

[15] Sanguin F Albiger N Betterle C Mian C Gatti R Rossi E Mantero F Scaroni C Diagnostic and therapeutic challenge in the management of a patient with ectopic adrenocorticotropin secretion. Journal of Endocrinological Investigation 2010 33 507–508. (10.1007/BF03346634) 20671411

[16] Verburg FA Anlauf M Mottaghy FM Karges W Somatostatin receptor imaging-guided pasireotide therapy in medullary thyroid cancer with ectopic adrenocorticotropin production. Clinical Nuclear Medicine 2015 40 e83–e84. (10.1097/RLU.0000000000000522) 24999678

[17] Davì MV Salgarello M Francia G Positive 68Ga-DOTATOC-PET/CT after cortisol level control during ketoconazole treatment in a patient with liver metastases from a pancreatic neuroendocrine tumor and ectopic Cushing syndrome. Endocrine 2015 49 566–567. (10.1007/s12020-014-0391-y) 25168486

[18] Barbot M Trementino L Zilio M Ceccato F Albiger N Daniele A Frigo AC Mardari R Rolma G Boscaro M, Second-line tests in the differential diagnosis of ACTH-dependent Cushing’s syndrome. Pituitary 2016 19 488–495. (10.1007/s11102-016-0729-y) 27236452

[19] Ceccato F Trementino L Barbot M Antonelli G Plebani M Denaro L Regazzo D Rea F Frigo AC Concettoni C, Diagnostic accuracy of increased urinary cortisol/cortisone ratio to differentiate ACTH-dependent Cushing’s syndrome. Clinical Endocrinology 2017 87 500–507. (10.1111/cen.13391) 28590513

[20] Antonelli G Artusi C Marinova M Brugnolo L Zaninotto M Scaroni C Gatti R Mantero F Plebani M Cortisol and cortisone ratio in urine: LC-MS/MS method validation and preliminary clinical application. Clinical Chemistry and Laboratory Medicine 2014 52 213–220. (10.1515/cclm-2013-0471) 24391193

[21] Ceccato F Albiger N Reimondo G Frigo AC Ferasin S Occhi G Mantero F Terzolo M Scaroni C Assessment of glucocorticoid therapy with salivary cortisol in secondary adrenal insufficiency. European Journal of Endocrinology 2012 167 769–776. (10.1530/EJE-12-0534) 23034783

[22] Antonelli G Ceccato F Artusi C Marinova M Plebani M Salivary cortisol and cortisone by LC-MS/MS: validation, reference intervals and diagnostic accuracy in Cushing’s syndrome. Clinica Chimica Acta: International Journal of Clinical Chemistry 2015 451 247–251. (10.1016/j.cca.2015.10.004) 26449783

[23] Davì MV Cosaro E Piacentini S Reimondo G Albiger N Arnaldi G Faggiano A Mantovani G Fazio N Piovesan A, Prognostic factors in ectopic Cushing’s syndrome due to neuroendocrine tumors: a multicenter study. European Journal of Endocrinology 2017 176 453–461. (10.1530/EJE-16-0809) 28183788

[24] Falhammar H Calissendorff J Höybye C Frequency of Cushing’s syndrome due to ACTH-secreting adrenal medullary lesions: a retrospective study over 10 years from a single center. Endocrine 2017 55 296–302. (10.1007/s12020-016-1127-y) 27699708 PMC5225211

[25] Albiger NM Occhi G Sanguin F Iacobone M Casarrubea G Ferasin S Mantero F Scaroni C Adrenal nodules in patients with Cushing’s disease: prevalence, clinical significance and follow-up. Journal of Endocrinological Investigation 2011 34 e204–e209. (10.3275/7349) 21088471

[26] Ueberberg B Tourne H Redmann A Walz MK Schmid KW Mann K Petersenn S Differential expression of the human somatostatin receptor subtypes sst1 to sst5 in various adrenal tumors and normal adrenal gland. Hormone and Metabolic Research 2005 37 722–728. (10.1055/s-2005-921092) 16372224

[27] Unger N Ueberberg B Schulz S Saeger W Mann K Petersenn S Differential expression of somatostatin receptor subtype 1–5 proteins in numerous human normal tissues. Experimental and Clinical Endocrinology and Diabetes 2012 120 482–489. (10.1055/s-0032-1314859) 22976314

[28] Ambrosini V Nanni C Fanti S The use of gallium-68 labeled somatostatin receptors in PET/CT imaging. PET Clinics 2014 9 323–329. (10.1016/j.cpet.2014.03.008) 25030395

[29] Hofman MS Lau WFE Hicks RJ Somatostatin receptor imaging with 68 Ga DOTATATE PET/CT: clinical utility, normal patterns, pearls, and pitfalls in interpretation. RadioGraphics 2015 35 500–516. (10.1148/rg.352140164) 25763733

[30] de Bruin C Hofland LJ Nieman LK van Koetsveld PM Waaijers AM Sprij-Mooij DM van Essen M Lamberts SW de Herder WW Feelders RA Mifepristone effects on tumor somatostatin receptor expression in two patients with Cushing’s syndrome due to ectopic adrenocorticotropin secretion. Journal of Clinical Endocrinology and Metabolism 2012 97 455–462. (10.1210/jc.2011-1264) 22090282 PMC3275368

[31] de Bruin C Feelders RA Waaijers AM van Koetsveld PM Sprij-Mooij DM Lamberts SW Hofland LJ Differential regulation of human dopamine D2 and somatostatin receptor subtype expression by glucocorticoids in vitro. Journal of Molecular Endocrinology 2009 42 47–56. (10.1677/JME-08-0110) 18852217

